# Novel Dual Mitochondrial and CD44 Receptor Targeting Nanoparticles for Redox Stimuli-Triggered Release

**DOI:** 10.1186/s11671-018-2445-1

**Published:** 2018-02-02

**Authors:** Kaili Wang, Mengjiao Qi, Chunjing Guo, Yueming Yu, Bingjie Wang, Lei Fang, Mengna Liu, Zhen Wang, Xinxin Fan, Daquan Chen

**Affiliations:** Collaborative Innovation Center of Advanced Drug Delivery System and Biotech Drugs in Universities of Shandong, School of Pharmacy, Yantai University, Yantai, China

**Keywords:** Mitochondrial targeting, CD44 receptor targeting, Redox sensitivity, Oligomeric hyaluronic acid (oHA), Multifunctional nanoparticles

## Abstract

In this work, novel mitochondrial and CD44 receptor dual-targeting redox-sensitive multifunctional nanoparticles (micelles) based on oligomeric hyaluronic acid (oHA) were proposed. The amphiphilic nanocarrier was prepared by (5-carboxypentyl)triphenylphosphonium bromide (TPP), oligomeric hyaluronic acid (oHA), disulfide bond, and curcumin (Cur), named as TPP-oHA-S-S-Cur. The TPP targeted the mitochondria, the antitumor drug Cur served as a hydrophobic core, the CD44 receptor targeting oHA worked as a hydrophilic shell, and the disulfide bond acted as a connecting arm. The chemical structure of TPP-oHA-S-S-Cur was characterized by ^1^HNMR technology. Cur was loaded into the TPP-oHA-S-S-Cur micelles by self-assembly. Some properties, including the preparation of micelles, morphology, redox sensitivity, and mitochondrial targeting, were studied. The results showed that TPP-oHA-S-S-Cur micelles had a mean diameter of 122.4 ± 23.4 nm, zeta potential − 26.55 ± 4.99 mV. In vitro release study and cellular uptake test showed that TPP-oHA-S-S-Cur micelles had redox sensibility, dual targeting to mitochondrial and CD44 receptor. This work provided a promising smart multifunctional nanocarrier platform to enhance the solubility, decrease the side effects, and improve the therapeutic efficacy of anticancer drugs.

## Background

In recent years, in order to achieve the effective treatment of cancer, many drug delivery systems [1] were studied to prepare multifunctional macromolecule polymer drug carriers with structural modifications which could take many advantages such as enhancing the bioavailability of drugs by reducing their degradation rate, improving cellular uptake, allowing targeting and control of drug release, and reducing side effects [2, 3].

Curcumin (Cur), a diphenolic compound, was isolated from a traditional Chinese medicine *Curcuma longa*. Compared with general anti-tumor drugs, Cur had wide anti-tumor effects on many tumors such as prostate, breast, and colon cancer, and that relatively lower cytotoxicity [4]. But its inadequate solubility, instability, and poor bioavailability extremely limit its clinical application [5, 6]. In order to improve the solubility and bioavailability of hydrophobic drugs, a lot of well-designed nanomaterials have been fabricated by incorporation of hydrophobic drugs and hydrophilic material oligomeric hyaluronic acid (oHA) [7]. We designed a kind of amphipathic polymer-drug conjugates as nanocarrier by loading curcumin into polymer micelles via self-assembly, which had good stability and can prolong the drug half-life in blood circulation. Micelles with small particle size can passively accumulate to solid tumor through EPR effect of tumor angiogenesis effectively to achieve a better treatment effect [8, 9].

oHA, a small molecule derived from the degradation of HA, possesses unique properties, such as good hydrophilicity, biocompatibility, stability in plasma, and targeting to CD44 receptor in the surface of cell [7, 10, 11], while CD44 receptors are overexpressed in lung cancer cell and breast cancer cells, compared with lower expression in the normal cells [12]. The tumor cells have the unique cellular signals, including glutathione (GSH, 2–10 mM), low pH, and some enzymes, in contrast to the normal human cells [13–20], while disulfide bonds have reduction sensitivity and could fracture under the action of reduced GSH in the cytoplasm [21, 22].

Mitochondria are the vital organelles for cell survival and play an important role in energy production and apoptotic pathways [23–27]. Therefore, mitochondria have been long deemed as the subcellular targets applied for cancer treatment in the previous reports [28]. Due to the higher membrane potential of mitochondria among cellular organelles, triphenylphosphine, triphenylmethylphosphonium, and other kinds of lipophilic cations can be easily enriched in the mitochondria through their lipid bilayer hydrophilic barrier [29, 30].

**Fig. 1:**
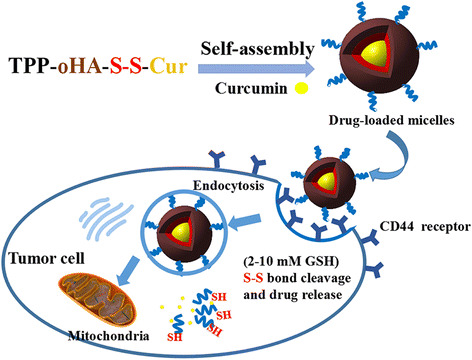
Schematic illustration of mitochondrial and CD44 receptor dual-targeting redox-sensitive nanoparticles

In this work, in order to enhance the solubility of Cur and enhance the specificity to cancer cells, polymer-drug conjugates, composed of oHA, TPP, disulfide bonds, and Cur, were designed and synthesized, which specifically targeted the CD44 receptor and mitochondria. Also, it had reduction sensitivity and can be used as a fluorescence marker. This multifunctional novel nanocarrier was named after (5-carboxypentyl)triphenylphosphonium bromide, oligomeric hyaluronic acid, disulfide bond and curcumin (TPP-oHA-S-S-Cur). Cur was loaded into TPP-oHA-S-S-Cur micelles via self-assembly in water [1, 31]. The polymeric TPP-oHA-S-S-Cur micelles encapsulated Cur (hereinafter denoted as Cur/TPP-oHA-S-S-Cur micelles) could enhance the therapeutic efficacy and the drug loading of curcumin, as well as reduce the side effects. After targeting the tumor tissues, the Cur/TPP-oHA-S-S-Cur micelles penetrated the cells through CD44-mediated endocytosis, followed by targeting the mitochondria and the disulfide bonds broke in response to high GSH (2–10 mM) leading to the rapid release of the drug (Fig. 1). Herein, we can propose a hypothesis that this novel mitochondrial and CD44 receptor dual-targeting redox-sensitive multifunctional nanoparticles will be a new platform for tumor targeting drug delivery system. After preparing micelles, some preliminary investigations about the characteristics of the Cur/TPP-oHA-S-S-Cur micelles were carried out in this study.

## Methods

### Materials

Curcumin (Cur, Shanghai Zhanyun Chemical Co. Ltd); oligomeric hyaluronic acid (oHA, Mn = 10 kDa, Shandong Freda Biological Engineering Co. Ltd.); 3,3′-dithiodipropionic acid was provided by the Adamas Reagent Co. Ltd. (Shanghai, China). (5-carboxypentyl)triphenylphosphoniumbromide (TPP), anhydrous tetrahydrofuran (THF), dimethyl sulfoxide (DMSO), triethylamine (TEA), 1-ethyl-(3-two methyl amino propyl) carbonized carbodiimide hydrochloride (EDC), and 4-dimethylaminopyridine (DMAP) were obtained from Aladdin Chemistry Co. Ltd. All other reagents were of analytical grade and supplied by Sinopharm Group Chemical Reagent Corp.

### Synthesis and Characterization of TPP-oHA-S-S-Cur

**Fig. 2:**
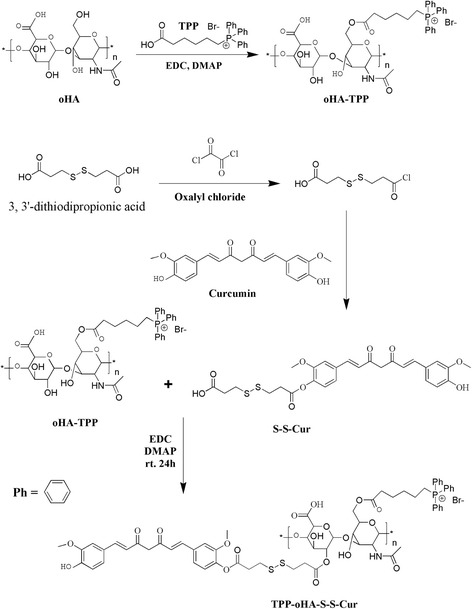
Synthetic route of TPP-oHA, S-S-Cur, and TPP-oHA-S-S-Cur

The multifunctional nanocarriers were synthesized with three steps, as shown in Fig. 2.

#### Step 1

TPP was combined with oHA via ester bond using a previously reported method [32]. First, TPP, EDC, and DMAP were dissolved in DMSO to react for 1 h at 60 °C. Then, oHA was dissolved in DMSO:H_2_O (*v*/*v* = 1:1) and added the above reaction into it for 8 h at room temperature. The reaction mixture was dialyzed with a dialysis bag (MWCO 2000 Da) in deionized water for 48 h to remove the impurities and lyophilized to obtain TPP-oHA polymers.

#### Step 2

Briefly, 3,3′-dithiodipropionic acid were dissolved in THF and activated by oxalyl chloride. Then, the mixture was reacted with Cur catalyzed by TEA. The obtained product was isolated by silica gel column chromatography to gain the pure product S-S-Cur.

#### Step 3

S-S-Cur, EDC, and DMAP were dissolved in DMSO and activated at 60 °C for 2 h, and then TPP-oHA was added in the above solution and reacted for 24 h at room temperature. The mixture was dialyzed in distilled water with dialysis tubing (MWCO 2000 Da) for 48 h, followed by lyophilizing to obtain the final product TPP-oHA-S-S-Cur.

The structures of TPP-oHA, S-S-Cur, and TPP-oHA-S-S-Cur were confirmed with ^1^HNMR (Advance Bruker 400M; Switzerland Bruker Company, Madison, WI, USA) using deuterated DMSO-d_6_, CD_3_Cl, and DMSO-D_6_: D_2_O (DMSO-d_6_: D_2_O = 1:1, *v*/*v*) as solvent, respectively.

### Preparation and Characterization of Micelles

The TPP-oHA-S-S-Cur micelles and oHA-Cur micelles were both prepared by dialysis methods [33]. Firstly, TPP-oHA-S-S-Cur (10 mg) and curcumin (1.5 mg) were dissolved in formamide (6 mL). Then, the mixture dialyzed in deionized water with dialysis bag (MWCO 2000 Da) in darkness for 24 h to remove the organic solvents. After that, dialyzed solution was centrifuged at 2500 rpm for 10 min to remove unloaded Cur. Finally, the micelle suspension was filtered through 0.45-μm syringe filter membranes. The single functional oHA-Cur micelles were obtained by the same method.

The particle size, zeta potential, and polydispersity index (P.I.) of Cur-loaded micelles were observed by Delsa Nano C (Beckman Coulter Inc.). The morphology of Cur-loaded micelles was monitored by transmission electron microscope (TEM, H-600; Hitachi, Tokyo, Japan). The stability of Cur-loaded micelles was carried out in PBS containing 20% FBS by the Delsa Nano C.

The entrapment efficiency (EE) and drug loading (DL) were measured by membrane filtration method. After filtering by 0.45 μm membrane, the obtained solution was measured by high-performance liquid chromatography (HPLC, Agilent Technologies) at 425 nm to obtain EE and DL. The equations to calculate EE and DL of the micelles were as follows:

$$ \mathrm{EE}\left(\%\right)=\mathrm{Amount}\ \mathrm{of}\ \mathrm{drug}\ \mathrm{in}\ \mathrm{micelles}/\mathrm{total}\ \mathrm{amount}\ \mathrm{of}\ \mathrm{feeding}\ \mathrm{drug}\times 100 $$

$$ \mathrm{DL}\left(\%\right)=\mathrm{Amount}\ \mathrm{of}\ \mathrm{drug}\ \mathrm{in}\ \mathrm{micelles}/\mathrm{amount}\ \mathrm{of}\ \mathrm{drug}\ \mathrm{loaded}\ \mathrm{micelles}\times 100 $$

### In Vitro Drug Release in the Presence of GSH

The intracellular GSH redox-responsiveness of Cur/TPP-oHA-S-S-Cur micelles was evaluated using the dialysis method. Four milliliters of the Cur/TPP-oHA-S-S-Cur micelle suspensions (50 μg curcumin/mL) was placed in a dialysis bag (MWCO 7000 Da) and dialyzed against 45 mL of PBS (pH 7.4, containing 45% fetal bovine serum and 0.5% Tween 80) with different GSH levels (0, 10, 2, and 10 mM). The samples in centrifuge tubes were kept at 37 °C in a shaker incubator (BS-2F, Changzhou, China) with 100 rpm. At predefined time intervals (0.25, 0.5, 1, 2, 4, 8, 12, 24, 48, 72, 96, and 120 h), 2 mL of release medium was taken from the centrifuge tubes and replenished with an equal volume of corresponding fresh media and the concentrations of Cur were analyzed by HPLC. Each experiment was run in triplicate.

### Cell Culture

MDA-MB-231 human breast carcinoma cells were cultured in Dulbecco’s modified Eagle’s medium (DMEM, Hyclone) containing 10% fetal bovine serum (FBS). CD44 receptors were high expressed in MDA-MB-231 cells, so MDA-MB-231 cell lines were selected and cultured in a humidified incubator at 37 °C with 5% CO_2_ atmosphere supplemented.

### In Vitro Cytotoxicity Assays

The in vitro cytotoxicity assay of TPP-oHA-S-S-Cur was evaluated by MTT method [32]. MDA-MB-231 cells (high-expressed CD44 receptor) were cultivated at a density of 1 × 10^4^ cells/well in 96-well plates. One hundred microliters of free Cur, Cur/oHA-Cur micelles, and Cur/TPP-oHA-S-S-Cur micelles including different concentrations of Cur (0.5, 1.25, 2.5, 5, 10, 20, and 40 μg/mL) was added to different wells, while PBS as control were added. After being incubated for 24 h, 100 μL of MTT solution (5 mg/mL) was added to each well and then incubated for 4 h. Then, MTT was replaced by 100 μL DMSO and put them in a shaker incubator to dissolve the formazan crystal. After incubating for 20 min, the absorbance was measured using a micro-plate reader (Thermo Fisher Scientific Co., Waltham, MA) at 570 nm.

### In Vitro Cellular Uptake and the Mitochondrial Localization

Qualitative analyses of cellular uptake of Cur/TPP-oHA-S-S-Cur micelles and Cur/oHA-Cur micelles were observed using fluorescence microscopy (Eclipse E400; Nikon Corporation, Tokyo, Japan). MDA-MB-231 cells were seeded into 24-well plates at a density of 5000 cells per well and incubated with DMEM (1 mL) containing 10% FBS and then cultured for 24 h at 37 °C in 5% CO_2_. After that, fresh culture medium (1 mL) with drug-loaded micelles (Cur/TPP-oHA-S-S-Cur micelles and Cur/oHA-Cur micelles) was added into each well. Cells were cultured with free Cur acted as control; the final Cur concentration was 20 μg/mL. Furthermore, the cells were incubated with drug for different time intervals. Then, the cells were washed three times with PBS and fixed with 4% paraformaldehyde for 15 min.

Additionally, to evaluate the CD44 targeting ability of Cur/TPP-oHA-S-S-Cur micelles, HA (2 mg/mL) was added to the medium as a control group to bind to the CD44 receptors.

Moreover, to label the cell mitochondria for locating Cur/TPP-oHA-S-S-Cur micelles and Cur/oHA-Cur micelles within cells, the cells were further incubated with a mitochondrial tracker (Mito-tracker Red CMXROS) for 15 min. Finally, the cells were washed three times and observed by fluorescence microscopy.

### Flow Cytometry

The quantitative analyses were measured using flow cytometry. MDA-MB-231 cells were seeded into six-well plates at a density of 10^5^ cells/well in 2 mL of media with 10% FBS and treated as the above description. After incubation with Cur/TPP-oHA-S-S-Cur micelles and Cur/oHA-Cur micelles (20 μg Cur/mL) for different time intervals, the cells were washed three times with PBS and trypsinized by 0.25% trypsin. Then, cells were centrifuged at 1500 rpm for 5 min. After resuspension in PBS (0.5 mL), the cells were analyzed by flow cytometry (EPICS XL, Beckman, USA).

### Statistical Analysis

The data were presented as means ± SD (*n* = 3). Moreover, SPSS software (ver. 20, USA) was used; the data were analyzed for significant differences at probability levels of *p < 0.05 (significant) using one-way analysis of variation (ANOVA).

## Results and Discussion

### Characterization of Carrier Materials TPP-oHA-S-S-Cur

The routes of synthesis of TPP-oHA, S-S-Cur and TPP-oHA-S-S-Cur were presented in Fig. 2.

**Fig. 3:**
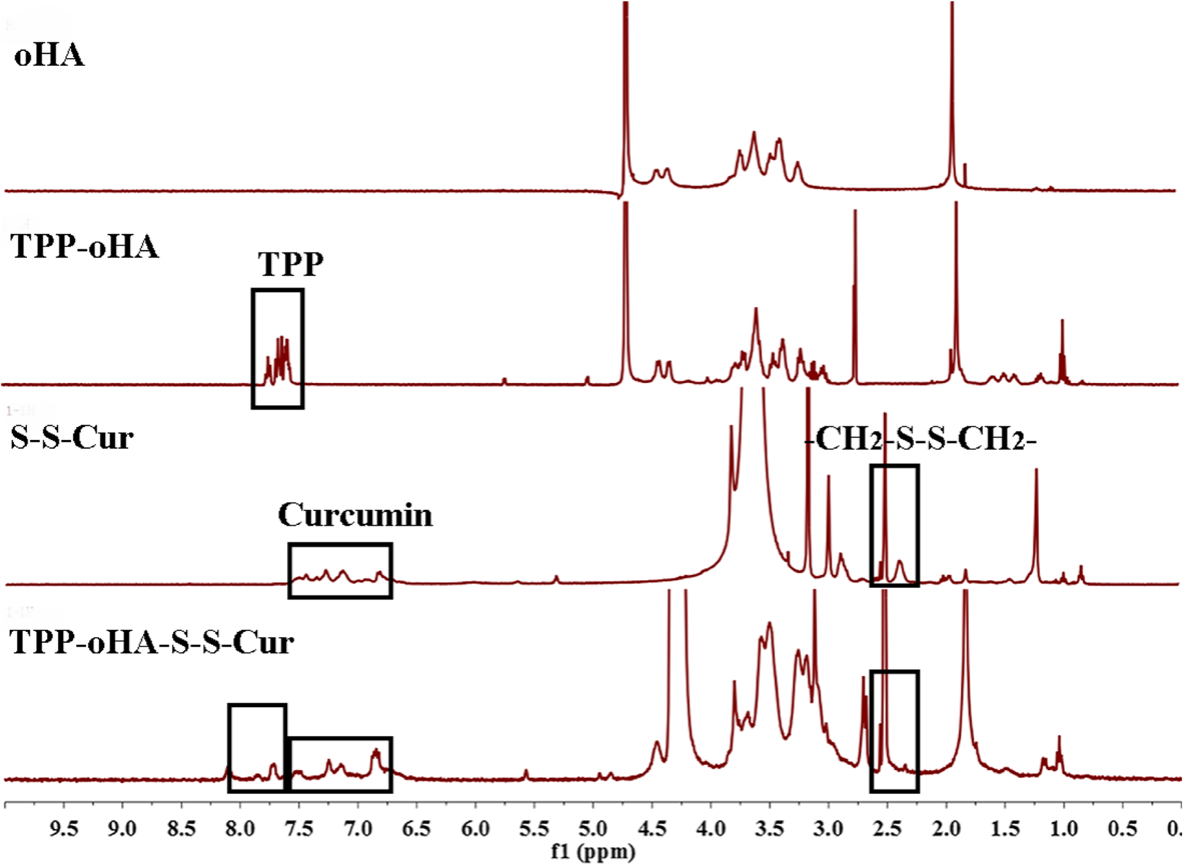
^1^HNMR spectra of oHA, TPP-oHA, S-S-Cur, and TPP-oHA-S-S-Cur

In addition, the ^1^HNMR spectra of oHA, TPP-oHA, S-S-Cur, and TPP-oHA-S-S-Cur were seen in Fig. 3. TPP-oHA was synthesized by TPP and oHA. Characteristic peaks of three benzene rings in TPP were seen at 7.570–7.717 ppm in the ^1^HNMR spectrum of TPP-oHA, which confirmed that TPP-oHA was synthesized successfully. Additionally, S-S-Cur was synthesized by 3,3′-dithio-dipropionic acid and Cur. Moreover, TPP-oHA-S-S-Cur was synthesized by TPP-oHA and S-S-Cur. The TPP in TPP-oHA-S-S-Cur was seen in the region between 7.570 and 7.717 ppm. The characteristic peaks of Cur were observed in the ^1^HNMR spectrum of S-S-Cur and TPP-oHA-S-S-Cur at 6.795–7.467 ppm, and the proton peak (-CH_2_-S-S-CH_2_-) of 3,3-dithiodipropionic acid was observed at 2.502 ppm in accordance with TPP-oHA-S-S-Cur. These results confirmed the successful synthesis of TPP-oHA-S-S-Cur.

### Preparation and Evaluation of TPP-oHA-S-S-Cur Micelles

The particle size, polydispersity index, zeta potentials, DL, and EE of the drug-loaded micelles are seen in Table 1. The average sizes of Cur/oHA-Cur micelles and Cur/TPP-oHA-S-S-Cur micelles were 145.5 ± 2.1 and 122.4 ± 4.6 nm, respectively. This might be because TPP changed the physiochemical properties of the surface of Cur/TPP-oHA-S-S-Cur micelles that lead to the differences of two micelles. And the DL and EE of Cur/TPP-oHA-S-S-Cur micelles were higher than the ordinary micelles that prepared by TPP-oHA. Moreover, the Cur/TPP-oHA-S-S-Cur micelles had stronger negative zeta potential (− 21.56 ± 1.46 mV) than that of Cur/oHA-Cur micelles (− 19.17 ± 0.55 mV), which indicated enhanced stability of TPP-oHA-S-S-Cur due to aggregation of micelle mainly caused by the presence of TPP.

**Table 1:** The physicochemical properties and stability of Cur/TPP-oHA-S-S-Cur micelles in PBS and PBS within FBS (*n* = 3)

Micelle	Cur/oHA-Cur micelles	Cur/TPP-oHA-S-S-Cur micelles
Particle size (nm)	145.5 ± 2.1	122.4 ± 4.6
Polydispersity Index	0.146 ± 0.37	0.132 ± 0.29
Zeta Potential (mV)	-19.17 ± 0.55	-21.56 ± 1.46
DL (%)	5.07 ± 4.6	5.45 ± 3.7
EE (%)	53.65 ± 6.2	59.62 ± 8.1
Time (h)	PBS	PBS + FBS
2	132.49 ± 7.8	133.45 ± 6.8
12	146.54 ± 11.5	158.87 ± 4.3
24	154.62 ± 6.7	160.27 ± 7.1

**Fig. 4:**
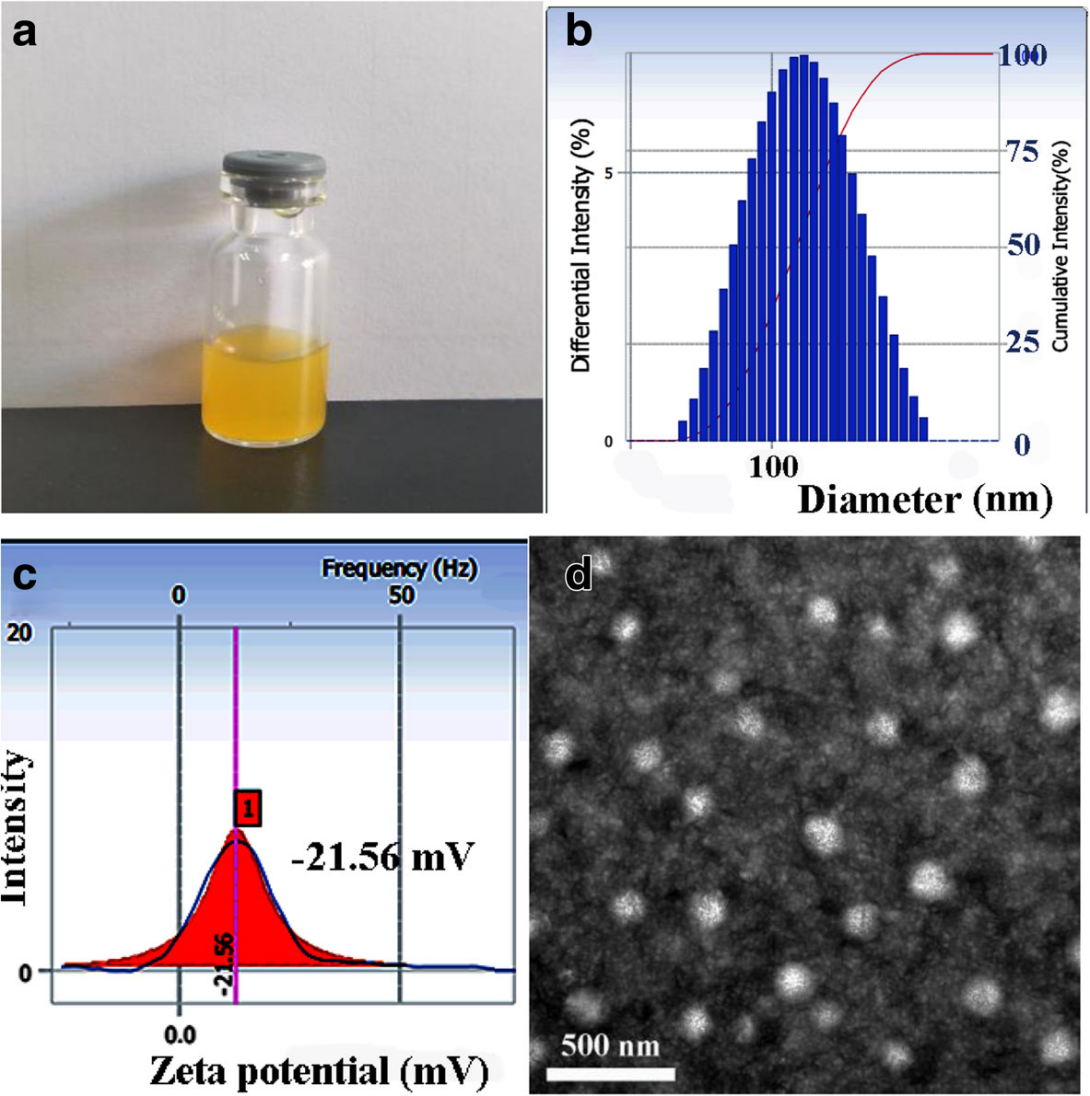
Appearance (**a**), particle size distribution (**b**), zeta potential (**c**), and TEM image (**d**) characterization of TPP-oHA-S-S-Cur micelles

In addition, the appearance, particle size distribution, zeta potential, and TEM image characterization of TPP-oHA-S-S-Cur micelles were shown in Fig. 4. The results revealed Cur/TPP-oHA-S-S-Cur micelles were approximately spherical (Fig. 4d), distributed homogeneously (Fig. 4b), and had an average diameter of approximately 122.4 ± 4.6 nm. Moreover, the polydispersity index of the Cur/TPP-oHA-S-S-Cur micelles was 0.132 smaller than 0.2, indicating the uniform of the micelles size, which were consistent with TEM (Fig. 4c).

As shown in Table 1, the particle size of Cur/TPP-oHA-S-S-Cur micelles was smaller in PBS than PBS containing FBS. From 2 to 24 h, the size had changed from 133.45 ± 6.8 to 160.27 ± 7.1 nm in PBS containing FBS. This phenomenon indicated that Cur/TPP-oHA-S-S-Cur micelles could maintain better stability in vivo.

### Study on Drug Release of Micelles In Vitro

**Fig. 5:**
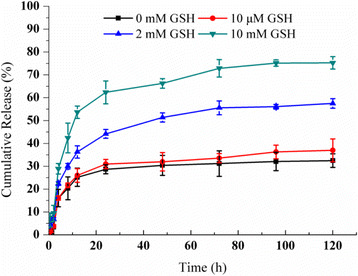
In vitro release of Cur-loaded TPP-oHA-S-S-Cur micelles at different GSH concentrations (*n* = 3)

The redox sensibility of TPP-oHA-S-S-Cur was studied by micelles in different GSH concentrations. Cur release from Cur/TPP-oHA-S-S-Cur micelles in the presence or absence of GSH was conducted to simulate the tumor environments. As shown in Fig. 5, in the medium without GSH, only 32.5% Cur was released from the TPP-oHA-S-S-Cur within 120 h, and addition of 10 μM GSH to the medium only resulted in slightly increase (37%). In contrast, the drug release of groups with 2 mM GSH and 10 mM GSH were 57.5 and 75.3%, respectively. This was verified that the drug release was increased with the GSH concentration. This result was attributed to disrupt disulfide bond of TPP-oHA-S-S-Cur, which indicated that the Cur/TPP-oHA-S-S-Cur micelles had reduction sensitivity.

### Cytotoxicity Studies

**Fig. 6:**
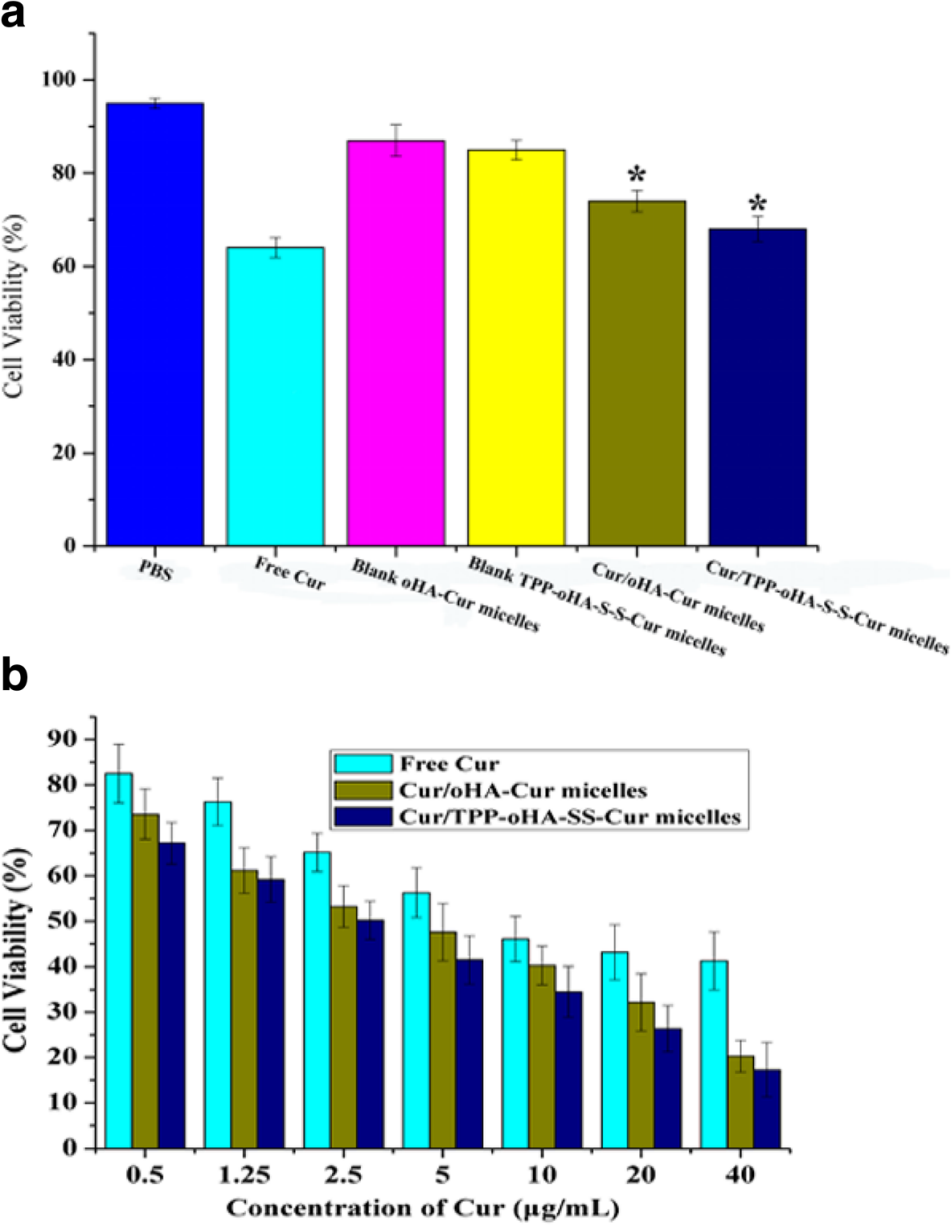
**a** Cytotoxicity of curcumin formulations after incubation for 24 h. Data represent the mean ± SD (*n* = 6). *p < 0.05, compared with the free Cur. **b** Cytotoxicity of Cur-loaded micelles with different concentrations of Cur (0.5, 1.25, 2.5, 5, 10, 20, and 40 μg/mL). The IC_50_ values of free Cur, Cur/oHA-Cur micelles, and Cur-TPP-oHA-SS-Cur micelles were 6.534, 5.092, and 3.871 μg/mL, respectively

The cytotoxicity of different curcumin formulations in MDA-MB-231 cells were studied by MTT assay. As shown in Fig. 6, different concentrations of curcumin formulations had different cell viability at 24 h. The micelles loaded with Cur were less toxic than the free Cur, which could be explained that the polymeric material greatly decreased the cell cytotoxicity. Meanwhile, Fig. 6b showed a concentration-dependent cell viability profile, while Cur/TPP-oHA-S-S-Cur micelles had the lowest cell viability than Cur/oHA-Cur micelles and other formulations, which could be explained that Cur/TPP-oHA-S-S-Cur micelles were easily to enter into the cells and release the drug under GSH effect. The IC_50_ values of free Cur, Cur/oHA-Cur micelles, and Cur-TPP-oHA-SS-Cur micelles in MDA-MB-231 cells were 6.534, 5.092, and 3.871 μg/mL, respectively, which exhibited better cellular cytotoxicity of Cur-TPP-oHA-SS-Cur micelles in MDA-MB-231 cells.

In addition, the blank micelles had certain toxicity (Fig. 6) because the Cur was linked to polymer by a chemical method. This would increase the drug loading capacity of micelles and then enhance the antitumor effect.

### Fluorescence Microscopy Images of the Cellular Uptake

**Fig. 7:**
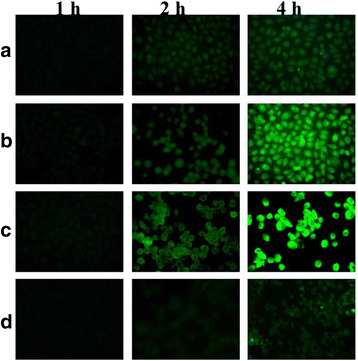
Fluorescence microscopy images of the cellular uptake of free Cur (**a**), oHA-Cur/ Cur-micelles (**b**), and TPP-oHA-S-S-Cur/Cur-micelles (**c**) at different time. **d** Cells treated with TPP-oHA-S-S-Cur/Cur-micelles in the presence of free HA (2 mg/mL), showing the completion effect of HA in MDA-MB-231 cells

The cellular uptake of free Cur, Cur/oHA-Cur micelles, and Cur/TPP-oHA-S-S-Cur micelles were studied by fluorescence microscope. In this work, Cur itself was not only an anticancer drug but also a green fluorescence probe. As seen in Fig. 7, both Cur/oHA-Cur micelles and Cur/TPP-oHA-S-S-Cur micelles showed good cellular uptake in cell lines and the fluorescence intensity was in direct proportion to time, while the fluorescence intensity was stronger at 4 h. Meanwhile, the fluorescence intensity of group treated with Cur/oHA-Cur micelles was stronger than the free Cur group at the same time points. This might because oHA-Cur with the ability of targeting CD44 receptor promoted the entry of drugs into cells. Furthermore, maybe thanks to the redox-sensitive ability of Cur/TPP-oHA-S-S-Cur micelles, its fluorescence signals were obviously higher than Cur/oHA-Cur micelles. By enhancing the Cur accumulation, it would be induced by the cytotoxicity and apoptotic of cancer cells, which were consistent with the results of cytotoxicity studies.

In addition, the fluorescence intensity of the Cur/TPP-oHA-S-S-Cur micelle group with HA was lower than that without HA group, most likely due to the fact that the free HA molecules preoccupied the CD44 receptors, which further demonstrated targeting ability of the TPP-oHA-S-S-Cur against CD44.

**Fig. 8:**
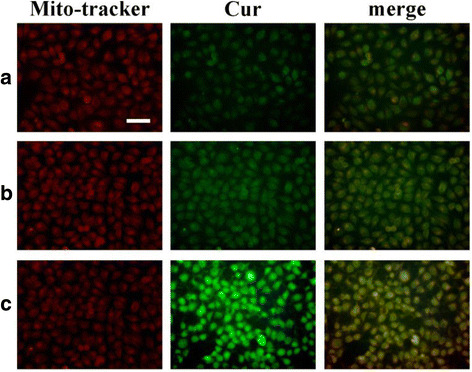
Mitochondrial localization. Mitochondrial localization was determined by staining with mitochondrial trackers in MCF-7 cells with the treatment of free Cur (**a**), oHA-Cur (**b**), and TPP-oHA-S-S-Cur (**c**); scale bar: 100 μm

Furthermore, the mitochondrial localization of TPP-oHA-S-S-Cur was confirmed in MDA-MB-231 cell lines by staining with Mito-tracker Red CMXRos (Fig. 8). The images revealed that the green fluorescence of Cur/TPP-oHA-S-S-Cur micelles nicely overlapped the red fluorescence of the mitochondrial tracker, which indicated that the drug could accumulate in the mitochondria of the cancer cells, and the TPP-oHA-S-S-Cur had mitochondrial targeting.

### Flow Cytometry

**Fig. 9:**
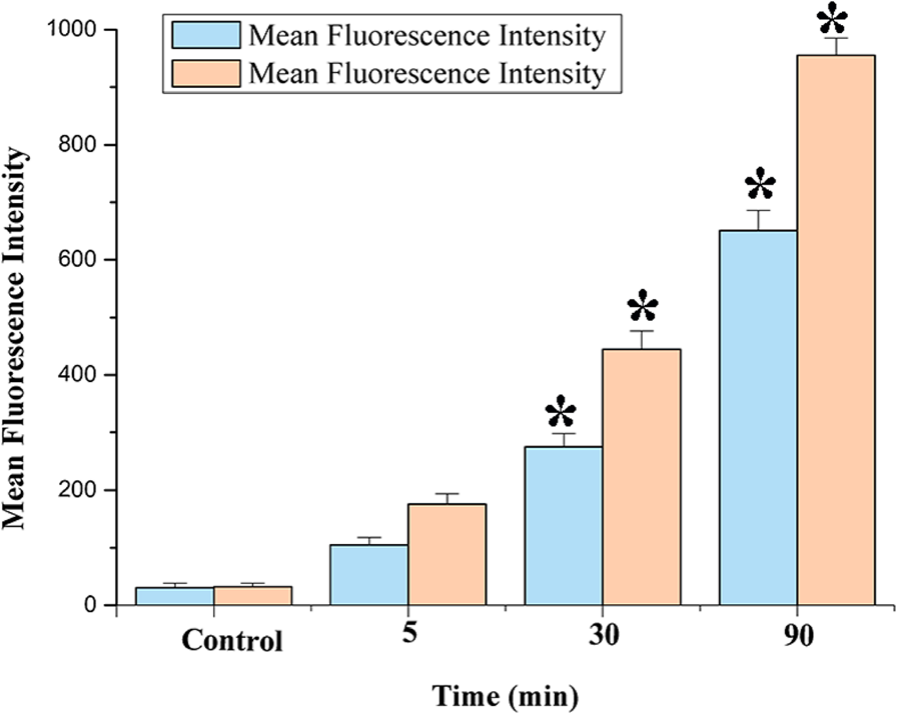
Fluorescence intensity of MDA-MB-231 cells incubated with different Cur formulations (20 μg Cur/mL). Data represented as mean ± SD (*n* = 3). *p < 0.05, one-way ANOVA

The mean fluorescent intensity (MFI) was measured with flow cytometry. As seen in Fig. 9, the MFI of MDA-MB-231 cells incubated with Cur/TPP-oHA-S-S-Cur micelles and Cur/oHA-Cur micelles was directly proportional to the administration time. Consistent with confocal microscopic observation, the MFI of cells treated with Cur/TPP-oHA-S-S-Cur micelles was significant higher than the Cur/oHA-Cur micelles group at the same time points (*p* < 0.05).

## Conclusions

In this study, to enhance the solubility and treatment effect of Cur, reduce the side effects of traditional therapy, and increase the tumor targeting of drug, we took redox-responsive disulfidebond as a connection arm and built a mitochondrial and CD44 receptor dual-targeting redox-responsive polymer-drug conjugates (TPP-oHA-S-S-Cur). The TPP targeted the mitochondria, antitumor drug Cur served as hydrophobic moiety, and the CD44 receptor targeting oHA acted as hydrophilic moieties. Cur, as a model drug, was loaded in the amphiphilic blockcopolymer and formed micelles via self-assembly.

This drug delivery system encapsulating Cur by a chemical method and a physical method not only improve its DL/EE but also increase its stability and blood circulation time, and it could achieve better tumor targeting. In vitro drug release tests showed that the disulfide bond of TPP-oHA-S-S-Cur broke by the effect of high GSH (2–10 mM) in the tumor cells, followed by the rapid release of the drug and then demonstrated its reduction-sensitive. The results of cellular uptake, mitochondrial localization, and cytotoxicity showed the Cur/TPP-oHA-S-S-Cur micelles had CD44 receptor-targeting ability and mitochondrial targeting ability. Next step, we will evaluate the antitumor activity of Cur/TPP-oHA-S-S-Cur micelles in vivo.

Moreover, this smart multifunctional nanocarrier platform developed in this study exhibited potential to be used for hydrophobic drug with significantly improved solubility, stability, and therapeutic efficacy. Meanwhile, this method provided a new idea for tumor treatment.

## Abbreviations

oHA: Oligomeric hyaluronic acid
S-S-Cur: Dithiodipropionic acid-curcumin
TPP: (5-Carboxypentyl)triphenyl-phosphonium bromide
